# Dietary intake of *trans* fatty acids and breast cancer risk in 9 European countries

**DOI:** 10.1186/s12916-021-01952-3

**Published:** 2021-03-30

**Authors:** Michèle Matta, Inge Huybrechts, Carine Biessy, Corinne Casagrande, Sahar Yammine, Agnès Fournier, Karina Standahl Olsen, Marco Lukic, Inger Torhild Gram, Eva Ardanaz, Maria-José Sánchez, Laure Dossus, Renée T. Fortner, Bernard Srour, Franziska Jannasch, Matthias B. Schulze, Pilar Amiano, Antonio Agudo, Sandra Colorado-Yohar, J. Ramón Quirós, Rosario Tumino, Salvatore Panico, Giovanna Masala, Valeria Pala, Carlotta Sacerdote, Anne Tjønneland, Anja Olsen, Christina C. Dahm, Ann H. Rosendahl, Signe Borgquist, Maria Wennberg, Alicia K. Heath, Dagfinn Aune, Julie Schmidt, Elisabete Weiderpass, Veronique Chajes, Marc J. Gunter, Neil Murphy

**Affiliations:** 1 Nutrition and Metabolism Branch, International Agency for Research on Cancer, 150 cours Albert Thomas, 69372 Lyon Cedex 08, France; 2 CESP “Health Across Generations”, INSERM, Univ Paris-Sud, UVSQ, Univ Paris-Saclay, Villejuif, France; 3 Gustave Roussy, Villejuif, France; 4 Department of Community Medicine, University of Tromsø, The Arctic University of Norway, Tromsø, Norway; 5 Navarra Public Health Institute, Pamplona, Spain; 6 IdiSNA, Navarra Institute for Health Research, Pamplona, Spain; 7 Centro de Investigación Biomédica en Red de Epidemiología y Salud Pública (CIBERESP), Madrid, Spain; 8 Escuela Andaluza de Salud Pública (EASP), Granada, Spain; 9 Instituto de Investigación Biosanitaria ibs.GRANADA, Granada, Spain; 10 Department of Preventive Medicine and Public Health, University of Granada, Granada, Spain; 11 Division of Cancer Epidemiology, German Cancer Research Centre (DFKZ), Heidelberg, Germany; 12 Department of Molecular Epidemiology, German Institute of Human Nutrition Potsdam-Rehbruecke, Nuthetal, Germany; 13 German Center for Diabetes Research (DZD), München-Neuherberg, Germany; 14 NutriAct - Competence Cluster Nutrition Research Berlin-Potsdam, Nuthetal, Germany; 15 Public Health Division of Gipuzkoa, BioDonostia Research Institute, Donostia-San Sebastian, Spain; 16 Unit of Nutrition and Cancer, Catalan Institute of Oncology - ICO, Nutrition and Cancer Group, Bellvitge Biomedical Research Institute - IDIBELL, L’Hospitalet de Llobregat, 08908 Barcelona, Spain; 17 Department of Epidemiology, Murcia Regional Health Council, IMIB-Arrixaca, Murcia, Spain; 18 Research Group on Demography and Health, National Faculty of Public Health, University of Antioquia, Medellín, Colombia; 19 Public Health Directorate, Asturias, Spain; 20 Cancer Registry and Histopathology Department, Provincial Health Authority (ASP 7), Ragusa, Italy; 21 Dipartimento Di Medicina Clinica e Chirurgia, Federici II University, Naples, Italy; 22 Cancer Risk Factors and Lifestyle Epidemiology Unit, Institute for Cancer Research, Prevention and Clinical Network – ISPRO, Florence, Italy; 23 Epidemiology and Prevention Unit, Fondazione IRCCS Istituto Nazionale dei Tumori, di Milano Via Venezian, 1, 20133 Milan, Italy; 24 Unit of Cancer Epidemiology, Città della Salute e della Scienza University-Hospital, Via Santena 7, 10126 Turin, Italy; 25 Danish Cancer Society Research Center, Copenhagen, Denmark; 26 Department of Public Health, Copenhagen University, Copenhagen, Denmark; 27 Department of Public Health, Aarhus University, Aarhus, Denmark; 28 Clinical Sciences Lund, Oncology, Lund University and Skåne University Hospital, Lund, Sweden; 29 Department of Oncology, Aarhus University Hospital, Aarhus University, Aarhus, Denmark; 30 Department of Public Health and Clinical Medicine, Section of Sustainable Health, Umeå University, Umeå, Sweden; 31 Department of Epidemiology and Biostatistics, Imperial College London, London, UK; 32 Department of Nutrition, Bjørknes University College, Oslo, Norway; 33 Department of Endocrinology, Morbid Obesity and Preventive Medicine, Oslo University Hospital Ullevål, Oslo, Norway; 34 Cancer Epidemiology Unit, Nuffield Department of Population Health, University of Oxford, Oxford, UK; 35 Office of the Director, International Agency for Research on Cancer, Lyon, France

**Keywords:** Industrial *trans* fatty acids, Ruminant *trans* fatty acids, Breast cancer, Diet

## Abstract

**Background:**

*Trans* fatty acids (TFAs) have been hypothesised to influence breast cancer risk. However, relatively few prospective studies have examined this relationship, and well-powered analyses according to hormone receptor-defined molecular subtypes, menopausal status, and body size have rarely been conducted.

**Methods:**

In the European Prospective Investigation into Cancer and Nutrition (EPIC), we investigated the associations between dietary intakes of TFAs (industrial *trans* fatty acids [ITFAs] and ruminant *trans* fatty acids [RTFAs]) and breast cancer risk among 318,607 women. Multivariable hazard ratios (HRs) and 95% confidence intervals (CIs) were estimated using Cox proportional hazards models, adjusted for other breast cancer risk factors.

**Results:**

After a median follow-up of 8.1 years, 13,241 breast cancer cases occurred. In the multivariable-adjusted model, higher total ITFA intake was associated with elevated breast cancer risk (HR for highest vs lowest quintile, 1.14, 95% CI 1.06–1.23; *P* trend = 0.001). A similar positive association was found between intake of elaidic acid, the predominant ITFA, and breast cancer risk (HR for highest vs lowest quintile, 1.14, 95% CI 1.06–1.23; *P* trend = 0.001). Intake of total RTFAs was also associated with higher breast cancer risk (HR for highest vs lowest quintile, 1.09, 95% CI 1.01–1.17; *P* trend = 0.015). For individual RTFAs, we found positive associations with breast cancer risk for dietary intakes of two strongly correlated fatty acids (Spearman correlation *r* = 0.77), conjugated linoleic acid (HR for highest vs lowest quintile, 1.11, 95% CI 1.03–1.20; *P* trend = 0.001) and palmitelaidic acid (HR for highest vs lowest quintile, 1.08, 95% CI 1.01–1.16; *P* trend = 0.028). Similar associations were found for total ITFAs and RTFAs with breast cancer risk according to menopausal status, body mass index, and breast cancer subtypes.

**Conclusions:**

These results support the hypothesis that higher dietary intakes of ITFAs, in particular elaidic acid, are associated with elevated breast cancer risk. Due to the high correlation between conjugated linoleic acid and palmitelaidic acid, we were unable to disentangle the positive associations found for these fatty acids with breast cancer risk. Further mechanistic studies are needed to identify biological pathways that may underlie these associations.

**Supplementary Information:**

The online version contains supplementary material available at 10.1186/s12916-021-01952-3.

## Background

Breast cancer is the most commonly diagnosed malignancy among women with over 2 million cases diagnosed globally in 2018 [1]. Despite being extensively studied, few established dietary risk factors for breast cancer have been identified [2]. The association between dietary fat intake and breast cancer risk has been a source of controversy with conflicting results reported in past decades [3–5]. Limited epidemiological evidence suggests that rather than total fat intake, types of fatty acids may diversely influence breast cancer risk [6, 7].

*Trans* fatty acids (TFAs) have been hypothesised to influence breast cancer risk [8]. TFAs can come from industrial processes generating industrial *trans* fatty acids (ITFAs), used in frying oils, margarines, and bakery products, or from ruminant *trans* fatty acids (RTFAs), from dairy and meat sources. Dietary intake of TFAs has been linked in experimental and observational studies to adiposity, insulin resistance, and systemic inflammation [9, 10], all risk factors for breast cancer [2, 11, 12]. However, the few prospective studies that have examined how dietary intakes of TFAs relate to breast cancer risk have generally reported null results [13]. These prior prospective studies were usually of relatively small size and generally did not examine the associations between TFAs and hormone receptor-defined molecular subtypes of breast cancer. Recently, in a case–control study nested within the European Prospective Investigation into Cancer and Nutrition Study (EPIC), higher plasma phospholipid levels of ITFAs were associated with a raised risk of oestrogen receptor-negative (ER−) breast cancer, but not overall breast cancer risk [14]. This result suggests that the relation between TFAs and breast cancer may differ according to hormone receptor subtype. A comprehensive and sufficiently powered examination of how dietary intakes of TFAs are associated with overall breast cancer and its molecular-defined subtypes is therefore warranted.

We investigated the association between dietary intakes of TFAs (ITFAs and RTFAs) with breast cancer risk in the EPIC study, an ongoing multinational cohort with more than 318,000 women. The large number of incident breast cancer cases (> 13,200 cases) affords high statistical power to examine the TFA associations across hormone receptor-defined molecular subtypes and body habitus.

## Methods

### Study population

EPIC is a multicentre cohort of 521,330 participants (mostly aged 35 years and older) who were recruited between 1992 and 2000, predominantly from the general population of 10 European countries (Denmark, France, Germany, Greece, Italy, Netherlands, Norway, Spain, Sweden, and the UK) [15, 16]. Written informed consent was provided by all study participants. Ethical approval for this study was provided by the International Agency for Research on Cancer and the institutional review boards of the local participating EPIC centres. The present analysis excluded men (*n* = 157,994), women from Greece (*n* = 15,239; excluded due to an ongoing data protection issue), women with prevalent cancers at any site (*n* = 19,853), those with missing diagnosis or censoring date (*n* = 2892), and those with missing dietary or lifestyle information (*n* = 6745). Our analysis therefore included 318,607 women.

### Assessment of dietary intake and other covariates

Dietary intake was assessed during the baseline enrolment visit (1992–2000) by country-specific instruments that were developed and validated within the various source populations in EPIC [15, 16]. Self-administered questionnaires were used in all centres, except in Spain and Ragusa (Italy), where data were collected during personal interviews. In Malmo (Sweden), a combined semi-quantitative food frequency questionnaire and 7-day dietary diary and diet interview was used. In order to estimate the intakes of individual fatty acids, the EPIC Nutrient Database (ENDB) was matched with the National Nutrient Database for Standard Reference of the United States (NNDSR; developed at the United States Department of Agriculture [USDA]) [17, 18]. To date, most of the national food composition databases of the ten respective EPIC countries do not contain nutritional values for specific dietary components such as fatty acid isomers that have been included in the NNDSR food composition tables. In addition, the USDA database includes a large number of food and recipe items from various countries and eating cultures (> 8000 food items) and used standard reference analytical methods to obtain the respective nutritional values [19]. The USDA database was matched with the EPIC food list to extend the ENDB database with extra food components, including dietary fatty acids. Specific foods and recipes that were not included in the USDA were decomposed into ingredients that were available in the USDA table. The fatty acid intakes reported in this manuscript were obtained through this extra USDA matching, and their quality has been confirmed through different quality controls. The first type of quality control includes the double-checking of the work performed by the three dietitians among each other. The second type of quality control includes the comparison between the nutrient values obtained through the ENDB procedures (matching with the national food composition databases) and this new USDA matching for the 28 food components that had already been matched with the EPIC food consumption data. The third type of quality control includes the comparison of the nutrients included in the extended EPIC database with nutritional biomarkers available in the nested case–control studies in EPIC. All these quality controls confirmed the validity of the data on fatty acids and their different isomers included in this manuscript (e.g. the correlation between TFAs derived from the dietary questionnaires and from plasma phospholipids was 0.53). ITFAs included elaidic acid and its isomers. For RTFA, the individual fatty acids included were palmiteaidic acid, conjugated linoleic acid, and vaccenic acid. Palmitelaidic acid could also be classified as an ITFA; however, in our population, its main sources were from dairy products.

Lifestyle questionnaires, administered at recruitment, were used as a source of information on educational attainment, smoking habits, alcohol intake, physical activity, reproductive and menstrual characteristics, and other variables.

### Follow-up and ascertainment of breast cancer

Incident cancer cases were identified using population cancer registries in Denmark, Italy, the Netherlands, Norway, Spain, Sweden, and the UK. In France and Germany, cancer cases were identified during follow-up from a combination of sources including health insurance records, cancer and pathology registries, and active follow-up directly through study participants or their next of kin. Incident breast cancer cases included invasive epithelial tumours at the primary site. Breast cancer cases were classified as ICD-10 code C50. Data on ER status was available for 9500 cases (1716 ER− and 7784 ER+) and on progesterone receptor (PR) for 7973 cases (2708 PR− and 5265 PR+). When stratified by positive or negative receptor status, there were 1259 ER− and PR− cases and 4830 ER+ and PR+ breast cancer cases. Immunohistochemical measurements of ER and PR expression were carried out in each EPIC centre. The following criteria were applied for a positive receptor status: ≥ 10% cells stained, any ‘plus system’ description, ≥ 20 fmol/mg, an Allred score of > 3, and IRS ≥ 2, or an H-score ≥ 10. Participants with ambiguous positive hormone receptor scores were excluded from analyses involving tumour receptor status (10% cells stained, = 20 fmol/mg, Allred score = 3, IRS ‘1–2’ or 2, H-score = e10). Further stratification by compilation of human epidermal growth factor receptor 2 (HER2) was made delimiting four categories: (1) ER− and PR− and HER2−, with 412 cases; (2) ER+ and PR+ and HER2+, with 349 cases; (3) ER− and PR− and HER2+, with 248 cases; and (4) ER+ and PR+ and HER2−, with 2174 cases.

### Statistical analyses

Hazard ratios (HRs) and 95% confidence intervals (CIs) for breast cancer risk were estimated using Cox proportional hazards regression models. Age was used as the time-scale in all models. Time at entry was age at recruitment. Exit time was age at whichever of the following came first: cancer diagnosis (except non-melanoma skin cancer), death, emigration, or last follow-up. Models were stratified by age at recruitment in 1-year categories and study centre.

Dietary estimates of TFAs were classified into quintiles or quartiles (for the analyses by hormonal receptor subtypes) based on the distribution of dietary intakes of fatty acid levels in all women. Statistical tests for trend were calculated using the ordinal quintile/quartile variable entered into the models as a continuous variable (primary method) and by using the quintile median values as a continuous variable (sensitivity analysis). Multivariable models were adjusted for the following variables, all assessed at recruitment: height (cm; continuous), education level (none and primary, technical or professional, secondary, higher education, and missing/unknown), body mass index (BMI, kg/m^2^; continuous), physical activity index (inactive, moderately inactive, moderately active, active and missing/unknown), energy intake (kcal/day; continuous), age at first birth and parity combined (nulliparous, first birth before age 30 years, 1–2 children; first birth before age 30 years, ≥ 3 children; first birth at age or after age of 30 years and missing/unknown), alcohol consumption (g/day; continuous), menopausal status (premenopausal, postmenopausal, perimenopausal, surgical postmenopausal bilateral ovariectomy), and smoking status (never, former, current, missing/unknown). Additional adjustment for menopausal hormone replacement therapy, age at menopause, breastfeeding, oral contraceptive use, and family history of breast cancer resulted in virtually unchanged HR estimates. False discovery rate correction was computed (*Q* value) for the overall breast cancer multivariable models using the Benjamini–Hochberg method [20]. In sensitivity analyses, we adjusted for total energy intake using the residuals method; mutually adjusted the total ITFA and RTFA models; adjusted the total ITFA and RTFA multivariable models for dietary intakes of saturated fatty acids (SFA), monounsaturated fatty acids (MUFAs), and polyunsaturated fatty acids (PUFA); and adjusted the total ITFA and RTFA multivariable models for the Mediterranean diet and the World Cancer Research Fund (WCRF) diet scores.

Analyses were also conducted according to hormonal receptor status (ER− and PR−, ER+ and PR+, ER, and PR) leading to another stratification compiling HER2 with four categories (ER− and PR− and HER2−; ER+ and PR+ and HER2+; ER− and PR− and HER2+; ER+ and PR+ and HER2−). Tests of heterogeneity of associations were carried out based on chi-square statistics, calculated as the deviation of logistic *β*-coefficients observed in each of the breast cancer subgroups relative to the overall *β*-coefficients. We also examined the association between dietary intakes of TFAs and breast cancer risk by menopausal status (premenopausal, postmenopausal) and BMI group (normal, overweight, obese), as prior evidence suggests that the fatty acid and breast cancer relationship may differ according to body size [21]. Interaction terms (multiplicative scale) between these variables and dietary intakes of TFAs were included in separate models, and the statistical significance of the cross-product terms was evaluated using likelihood ratio tests. Heterogeneity across countries was explored using a meta-analytic approach [22].

Statistical tests were all two-sided, and a *P* value of < 0.05 was considered statistically significant. Analyses were conducted using Stata version 14.2 (StataCorp, College Station, TX, USA).

## Results

During a median follow-up of 8.1 years, 13,241 malignant breast cancer cases were diagnosed. Baseline characteristics of study participants are summarised in Table 1 by dietary intakes of total ITFA and RTFA and for breast cancer cases and non-cases in Additional file 1: Table S1. Compared with the non-cases, breast cancer cases were older with a greater proportion of postmenopausal women. Breast cancer cases reported higher alcohol consumption, were less physically active, and were more likely to have used hormone replacement therapy. The Spearman correlation matrix for dietary intake of the different TFAs is presented in Additional file 1: Table S2. Modest correlations were found between individual TFAs, with the exception of a high correlation (*r* = 0.77) found between the RTFAs, palmitelaidic acid, and conjugated linoleic acid. Food group sources of conjugated linoleic acid and elaidic acid (the predominant ITFA) are presented in Additional file 1: Table S3.

**Table 1:** Characteristics of study participants by dietary intake of total industrial *trans* fatty acid and total ruminant *trans* fatty acid

	Total industrial *trans* fatty acid intake, median (IQR)		Total ruminant *trans* fatty acid intake, median (IQR)	
	Quintile 1	Quintile 5	Quintile 1	Quintile 5
Age at recruitment, years	51.2 (44.0–57.2)	51.2 (43.8–58.5)	53.0 (48.3–59.1)	50.1 (44.8–57.1)
Follow-up, years	14.9 (13.8–16.3)	15.9 (14.1–17.3)	16.1 (14.1–17.5)	14.8 (12.2–15.2)
Weight, kg	63.5 (57.0–71.1)	64.1 (58.0–72.0)	66 (59.5–74.0)	61.4 (55.7–68.6)
Height, cm	159.5 (155.0–164)	164 (160.0–168.1)	163.0 (158.2–167.5)	162.4 (158.2–167.0)
Body mass index (BMI), kg/m^2^	24.9 (22.3–28.2)	23.8 (21.6–26.6)	24.8 (22.4–28.0)	23.1 (21.1–25.8)
Number of full-term pregnancies	1.0 (1.0–1.0)	1.0 (1.0–1.0)	1.0 (1.0–1.0)	1.0 (1.0–1.0)
Ever use oral contraceptives (%)				
Yes	31,013 (48.8)	36,167 (61.8)	31,779 (53.1)	40,973 (65.0)
Age at first birth combinations (%)				
Nulliparous	8374 (13.1)	9642 (15.1)	7700 (12.1)	9773 (15.3)
Age at first birth < 30 (1–2 children)	29,686 (46.6)	24,325 (38.1)	27,918 (43.8)	28,447 (44.6)
Age at first birth < 30 (3< children)	16,456 (25.8)	15,082 (23.6)	17,540 (27.5)	14,536 (22.8)
Age at first birth ≥ 30	7486 (11.7)	7003 (10.9)	5672 (8.9)	7655 (12.0)
Ever use hormone replacement therapy for menopause (%)				
Yes	13,345 (48.9)	15,832 (54.9)	18,372 (54.9)	16,432 (65.2)
Ever breastfed (%)				
Yes	44,205 (71.8)	37,254 (74.0)	43,305 (78.9)	39,427 (66.6)
Menopausal status (%)				
Premenopausal	24,076 (37.7)	21,332 (33.4)	15,837 (24.8)	23,139 (36.3)
Postmenopausal	26,710 (41.9)	28,886 (45.3)	34,109 (53.5)	26,157 (41.0)
Age at menopause, years	48.6 (46.0–52.0)	48.6 (46.0–52.0)	48.7 (46.0–52.0)	48.9 (46.0–52.0)
Alcohol intake (g/day)				
None	16,483 (25.8)	8166 (12.8)	12,376 (19.4)	7330 (11.5)
> 60 g/day	7193 (11.2)	2908 (4.5)	5138 (8.0)	5521 (8.6)
Total dietary energy intake (kcal/day)	1779 (1835–2527)	2155 (1835–2527)	1667 (1370–2008)	2305 (1960–2700)
Education status (%)				
None and primary school	29,078 (45.6)	15,907 (24.9)	25,398 (39.8)	10,750 (16.8)
Higher education	12,360 (19.4)	12,291 (19.2)	9581 (15.0)	20,510 (32.2)
Physical activity (%)				
Inactive	20,789 (32.6)	11,591 (18.1)	13,776 (21.6)	13,467 (21.1)
Active	6703 (10.5)	11,457 (17.9)	12,725 (19.9)	7922 (12.4)
Smoking status (%)				
Never	38,253 (60.0)	32,130 (50.4)	33,578 (52.6)	38,912 (61.1)
Current	12,300 (19.3)	15,844 (24.8)	15,468 (24.2)	9095 (14.2)

*IQR* interquartile range

### Dietary industrial *trans* fatty acid (ITFA) intake and breast cancer risk

In the multivariable model, higher dietary intake of total ITFAs was associated with elevated breast cancer risk (HR for highest vs lowest quintile, 1.14, 95% CI 1.06–1.23; *P* trend = 0.001) (Table 2). Higher breast cancer risk for total ITFA intake was found from the second quintile onwards (intakes ≥ 0.54 g/day). For individual ITFAs, a positive association was found between dietary intakes of elaidic acid and breast cancer risk (HR for highest vs lowest quintile, 1.14, 95% CI 1.06–1.23; *P* trend = 0.001) (Table 2). In analyses by tumour hormonal receptor status, there was little evidence of statistical heterogeneity (Table 3; Additional file 1: Tables S4-S6), although statistically significant positive associations were found for elaidic acid and total ITFAs with ER+/PR+ breast cancer (total ITFAs: HR for highest vs lowest quartile, 1.14, 95% CI 1.02–1.28; *P* trend = 0.009; elaidic acid: HR for highest vs lowest quartile, 1.14, 95% CI 1.01–1.27; *P* trend = 0.007), but not for ER−/PR− breast cancer (total ITFAs: HR for highest vs lowest quartile, 1.08, 95% CI 0.87−1.33; *P* trend = 0.48; elaidic acid: HR for highest vs lowest quartile, 1.08, 95% CI 0.87−1.34; *P* trend = 0.49) (Table 3). Similarly, when human HER2 status was further taken into consideration, more consistent positive associations were found for ITFAs with ER+/PR+/HER2− breast cancer than the ER+/PR+/HER2+ subtype (Additional file 1: Table S6).

**Table 2:** Associations between dietary intake of *trans* fatty acids and breast cancer risk

		Cases/participants	Intake range (mg/day)	Basic^§^	Multivariable^†^
				HR (95% CI)	HR (95% CI)
Total industrial *trans* fatty acids^a^	Q1	2324/63,722	< 544	1 (reference)	1 (reference)
	Q2	2674/63,721	544–< 973	1.10 (1.04–1.17)	1.10 (1.04–1.17)
	Q3	2692/63,722	973–< 1520	1.11 (1.04–1.18)	1.12 (1.05–1.20)
	Q4	2780/63,721	1520–< 2535	1.13 (1.06–1.21)	1.15 (1.07–1.23)
	Q5	2771/63,721	≥ 2535	1.11 (1.04–1.19)	1.14 (1.06–1.23)
	*P* trend			0.009	0.001
	*Q* value				0.002
Elaidic acid	Q1	2323/63,722	< 506	1 (reference)	1 (reference)
	Q2	2651/63,721	506–< 924	1.09 (1.03–1.16)	1.10 (1.03–1.17)
	Q3	2719/63,722	924–< 1455	1.12 (1.05–1.19)	1.13 (1.06–1.20)
	Q4	2771/63,721	1455–< 2470	1.14 (1.07–1.22)	1.16 (1.08–1.24)
	Q5	2777/63,721	≥ 2470	1.11 (1.04–1.19)	1.14 (1.06–1.23)
	*P* trend			0.005	0.001
	*Q* value				0.002
Total ruminant *trans* fatty acids^b^	Q1	2961/63,724	< 13.58	1 (reference)	1 (reference)
	Q2	2406/63,726	13.58–< 26.41	1.04 (0.98–1.11)	1.03 (0.97–1.10)
	Q3	2499/63,715	26.41–< 49.03	1.05 (0.98–1.12)	1.03 (0.97–1.10)
	Q4	2629/63,721	49.03–< 86.31	1.10 (1.03–1.18)	1.08 (1.01–1.16)
	Q5	2746/63,721	≥ 86.31	1.11 (1.04–1.19)	1.09 (1.01–1.17)
	*P* trend			0.001	0.015
	*Q* value				0.022
Palmitelaidic acid	Q1	3031/63,722	< 1.28	1 (reference)	1 (reference)
	Q2	2698/63,698	1.28–< 2.98	1.04 (0.98–1.10)	1.03 (0.97–1.09)
	Q3	2389/63,723	2.98–< 6.56	1.02 (0.96–1.09)	1.01 (0.95–1.08)
	Q4	2473/63,715	6.56–< 18.01	1.08 (1.01–1.15)	1.07 (1.00–1.14)
	Q5	2650/63,721	≥ 18.01	1.09 (1.02–1.17)	1.08 (1.01–1.16)
	*P* trend			0.007	0.028
	*Q* value				0.034
Conjugated linoleic acid	Q1	2953/63,725	< 10.18	1 (reference)	1 (reference)
	Q2	2394/63,720	10.18–< 19.25	1.03 (0.97–1.09)	1.02 (0.96–1.09)
	Q3	2494/63,720	19.25–< 35.63	1.07 (1.00–1.14)	1.05 (0.99–1.13)
	Q4	2632/63,721	35.63–< 65.32	1.13 (1.05–1.21)	1.11 (1.03–1.19)
	Q5	2768/63,721	≥ 65.32	1.14 (1.06–1.22)	1.11 (1.03–1.20)
	*P* trend			< 0.001	0.001
	*Q* value				0.002
Vaccenic acid	Q1/Q2^c^	5701/130,242	< 0.07	1 (reference)	1 (reference)
	Q3	2434/60,976	0.07–< 0.08	1.03 (0.96–1.11)	1.03 (0.96–1.11)
	Q4	2646/63,675	0.08–< 2.24	1.04 (0.97–1.11)	1.03 (0.96–1.11)
	Q5	2460/63,714	≥ 2.24	1.04 (0.96–1.12)	1.02 (0.95–1.10)
	*P* trend			0.34	0.51
	*Q* value				0.51

*HR* hazard ratio, *CI* confidence interval

^§^Stratified by study centre and age (in 1-year categories)

^†^Stratified by study centre and age (in 1-year categories) and adjusted for total energy intake (kcal/day; continuous), body mass index (kg/m^2^; continuous), height (cm; continuous), alcohol consumption (g/day; continuous), education level (none and primary, technical or professional and secondary, higher education), age at first birth and parity combined (nulliparous, first birth before age 30 years, 1–2 children; first birth before age 30 years, ≥ 3 children; first birth ≥ 30 years), physical activity (inactive, moderately inactive, moderately active, and active), menopausal status (premenopausal, postmenopausal, perimenopausal, surgical postmenopausal bilateral ovariectomy), and smoking status (never, former, current smoker, unknown)

^a^Total industrial *trans* fatty acids included 18:1n-9 t, 18:2n-6tt

^b^Total ruminant *trans* fatty acids included 16:1n-9 t, 18:1n-7t, conjugated linoleic acid

^c^Quintiles 1 and 2 merged due to extreme low intake values in these groups

**Table 3:** Associations between dietary intake of *trans* fatty acids and molecular subtypes of breast cancer risk

		ER− and PR−	ER+ and PR+	*P*_heterogeneity_
		***n*** = 1259 HR (95% CI)	***n*** = 4830 HR (95% CI)	
Total industrial *trans* fatty acids^a^	Q1	1 (reference)	1 (reference)	
	Q2	0.99 (0.84–1.18)	1.13 (1.04–1.23)	
	Q3	1.02 (0.85–1.22)	1.18 (1.07–1.29)	
	Q4	1.08 (0.87–1.33)	1.14 (1.02–1.28)	
	*P* trend	0.48	0.009	0.55
Elaidic acid	Q1	1 (reference)	1 (reference)	
	Q2	0.99 (0.83–1.17)	1.12 (1.03–1.22)	
	Q3	1.01 (0.84–1.21)	1.19 (1.08–1.31)	
	Q4	1.08 (0.87–1.34)	1.14 (1.01–1.27)	
	*P* trend	0.49	0.007	0.52
Total ruminant *trans* fatty acids^b^	Q1	1 (reference)	1 (reference)	
	Q2	1.08 (0.89–1.32)	1.02 (0.92–1.13)	
	Q3	1.13 (0.92–1.39)	1.01 (0.91–1.12)	
	Q4	1.07 (0.85–1.34)	1.11 (0.99–1.25)	
	*P* trend	0.63	0.055	0.64
Palmitelaidic acid	Q1	1 (reference)	1 (reference)	
	Q2	0.91 (0.76–1.09)	1.01 (0.92–1.11)	
	Q3	1.05 (0.86–1.28)	1.07 (0.96–1.18)	
	Q4	1.03 (0.84–1.26)	1.09 (0.98–1.21)	
	*P* trend	0.46	0.07	0.84
Conjugated linoleic acid	Q1	1 (reference)	1 (reference)	
	Q2	1.09 (0.90–1.33)	0.99 (0.89–1.09)	
	Q3	1.11 (0.90–1.38)	1.01 (0.90–1.12)	
	Q4	1.12 (0.89–1.41)	1.10 (0.98–1.24)	
	*P* trend	0.38	0.056	0.90
Vaccenic acid	Q1	1 (reference)	1 (reference)	
	Q2	0.98 (0.76–1.26)	1.08 (0.94–1.23)	
	Q3	0.90 (0.71–1.14)	1.14 (1.01–1.29)	
	Q4	0.92 (0.72–1.17)	1.09 (0.96–1.23)	
	*P* trend	0.39	0.26	0.20

Stratified by study centre and age (in 1-year categories) and adjusted for total energy intake (kcal/day; continuous), body mass index (kg/m^2^; continuous), height (cm; continuous), alcohol consumption (g/day; continuous), education level (none and primary, technical or professional and secondary, higher education), age at first birth and parity combined (nulliparous, first birth before age 30 years, 1–2 children; first birth before age 30 years, ≥ 3 children; first birth ≥ 30 years), physical activity (inactive, moderately inactive, moderately active, and active), menopausal status (premenopausal, postmenopausal, perimenopausal, surgical postmenopausal bilateral ovariectomy), and smoking status (never, former, current smoker, unknown)

*ER− and PR−* oestrogen receptor-negative/progesterone receptor-negative, *ER+ and PR+* oestrogen receptor-positive and progesterone receptor-positive, *HR* hazard ratio, *CI* confidence interval

^a^Total industrial *trans* fatty acids included 18:1n-9t, 18:2n-6tt

^b^Total ruminant *trans* fatty acids included 16:1n-9t, 18:1n-7t, conjugated linoleic acid

### Dietary ruminant *trans* fatty acid (RTFA) intake and breast cancer risk

In the multivariable model, dietary intake of total RTFA was positively associated with breast cancer risk (HR for highest vs lowest quintile, 1.09, 95% CI 1.01–1.17; *P* trend = 0.015) (Table 2). Among individual RTFAs, higher dietary intake of palmitelaidic acid (HR for highest vs lowest quintile, 1.08, 95% CI 1.01–1.16; *P* trend = 0.028) and conjugated linoleic acid was associated with greater breast cancer risk (HR for highest vs lowest quintile, 1.11, 95% CI 1.03–1.20; *P* trend = 0.001). No association was found between intake of vaccenic acid and breast cancer risk (HR for highest vs lowest quintiles, 1.02, 95% CI 0.95–1.10; *P* trend = 0.51). For RTFAs, there was little evidence of heterogeneity by hormonal receptor status (Table 3 and Additional file 1: Tables S4-S6).

### Subgroup and sensitivity analyses

In subgroup analyses, there was no heterogeneity for the associations between total ITFAs and RTFAs with breast cancer risk by BMI group, menopausal status (*P* heterogeneities ≥ 0.18; Additional file 1: Tables S7 and S8), and country (*I*^2^ = 0%, *P* heterogeneities > 0.9; Additional file 1: Figures S1 and S2). Similar associations were found when we adjusted for total energy intake using the residuals method (Additional file 1: Table S9); mutually adjusted the total ITFA and RTFA models (Additional file 1: Table S10); additionally adjusted the total ITFA and RTFA models for dietary intakes of SFA, MUFA, and PUFA (Additional file 1: Table S11); and additionally adjusted the total ITFA and RTFA models for the Mediterranean or WCRF diet scores (Additional file 1: Table S12). Similar tests for trend across dietary intake groups were found when the quintile median values were used as a continuous variable (Additional file 1: Table S13).

## Discussion

In this large multinational European study, we found that higher dietary intakes of total ITFAs and RTFAs were associated with greater breast cancer risk. For ITFAs, a positive association was found for intake of elaidic acid, with no heterogeneity found across breast cancer hormone receptor-defined molecular subtypes. For RTFAs, higher intake of dietary conjugated linoleic acid was unexpectedly associated with greater breast cancer risk, although intake of conjugated linoleic acid in our population was strongly correlated with intake of palmitelaidic acid, which was also positively associated with breast cancer risk. For all TFAs, similar associations with breast cancer risk were found according to menopausal status and BMI group.

To our knowledge, this is the first prospective study to find that higher dietary intake of ITFAs was associated with raised breast cancer risk. We found a similar magnitude positive association for dietary intake of elaidic acid, the predominant ITFA. Previously, an analysis in the VITamins And Lifestyle (VITAL) cohort reported a suggestive positive association for the intake of elaidic acid that did not reach the threshold of statistical significance [23], while other perspective studies found no evidence of an association between ITFA intake and risk of breast cancer [24, 25]. Our positive association for dietary intake of ITFAs with overall breast cancer risk is concordant with findings from a nested case–control study in the French E3N study, in which higher serum phospholipid ITFA levels were associated with elevated breast cancer risk (odds ratio [OR] for highest vs lowest quintile, 1.75, 95% CI 1.08–2.83; *P* trend = 0.018) [26]. In another previous analysis in the EPIC study, a similar positive association was found between serum ITFA levels and breast cancer risk, but only for ER− tumours (OR for highest vs lowest tertile, 2.01, 95% CI 1.03–3.90; *P* trend = 0.047) [14]. In contrast, we found no heterogeneity in the association between total ITFA intake and breast cancer risk according to hormone receptor-defined molecular subtypes. Collectively, evidence from most of these European studies supports a positive relationship between dietary intakes of ITFAs and breast cancer risk. However, further studies are required to understand the heterogeneity of this relationship across molecular subtypes of breast cancer defined by tumour hormonal receptor status.

Prior experimental evidence linking ITFAs with breast cancer is limited. Elaidic acid has been shown to modulate hepatic lipogenesis through upregulating the SREBP-1 pathway [27]. However, further mechanistic studies are needed to better understand the possible detrimental health effects of ITFAs in relation to breast cancer development.

ITFAs, created when fats and oils are partially hydrogenated during industrial processing, are found in fast foods, industrially produced products and snacks, deep fried foods, baked goods, and ultra-processed foods. Since the 1990s, ITFA content in popular food products found in Europe has declined [28, 29], and many countries do not limit their content in food products [30]. In 2019, the European Union (EU) set new recommendations for ITFA intake, in accordance with those from the World Health Organization (WHO), for foods to be largely free of industrial *trans* fats by 2023 [31, 32]. These recommendations set a threshold of no more than 2 g per 100 g on ITFA products, and country members have until 2021 to implement these changes [33]. It is of note, however, that in our data, raised breast cancer risk was found at relatively low total dietary ITFA intake levels (≥ 0.54 g per day), when compared with participants with intake below this level. Consequently, adherence to the new EU regulations on IFTA content of foods may have minimal impact on the dietary ITFA intake and breast cancer relationship. However, further high-quality studies are required to confirm the positive association we found between dietary ITFA intake and breast cancer risk.

We also found an unexpected association between higher dietary intake of total RTFAs and elevated breast cancer risk. This result was driven by positive associations of similar magnitude for conjugated linoleic acid and palmitelaidic acid, with vaccenic acid being unrelated to breast cancer risk. Conjugated linoleic acid comprises a family of positional and geometric isomers of linoleic acid and is mostly found in meat and dairy products derived from ruminants. Several experimental studies have demonstrated protective effects of conjugated linoleic acid in the mammary gland at pharmacological doses [34–38]. In rodent models, conjugated linoleic acid had anti-proliferative effects in mammary tumorigenesis [37]. While in human breast tissue and in vitro studies, conjugated linoleic acid has been shown to induce apoptosis and inhibit breast cancer cellular proliferation via ER-mediated pathways [35, 38]. Finally, specific conjugated linoleic acid isomers have been shown to regulate mammary tumour growth, inducing expression of apoptotic genes and inhibiting cellular growth [34]. This experimental evidence, however, is not supported by prospective epidemiological evidence. Analyses in the Swedish Mammography Cohort and Melbourne Collaborative Cohort Study found no association between dietary intake of conjugated linoleic acid and breast cancer risk [39, 40]. An analysis in the Netherlands Cohort Study on Diet and Cancer, similar to our result, found a positive association between conjugated linoleic acid and breast cancer risk (relative risk for highest vs lowest quintile, 1.24, 95% CI 0.91–1.69; *P* trend = 0.02) [41]. In our data, dietary intake of conjugated linoleic acid was strongly correlated with intake of palmitelaidic acid (Spearman correlation *r* = 0.77), an RTFA sourced from hydrogenated vegetable oils and dairy foods, for which we also found a positive association with breast cancer risk; consequently, we are unable to separate the positive associations found for these fatty acids. Overall, prospective epidemiological data provide little evidence to support the anti-tumorigenic effects of conjugated linoleic acid on breast cancer development found in experimental studies.

This was the largest study to comprehensively examine the association between dietary intakes of TFAs and breast cancer. The large sample size and high-quality epidemiological and clinical data allowed us to examine the association by tumour molecular subtypes and according to menopausal status and BMI group. A limitation of our study is that dietary intake was measured once at baseline (using questionnaires) and consequently may be subject to random measurement error and not reflective of longer-term habits; any such bias would likely lead to an underestimation of true associations. In addition, like all studies using self-reported dietary intake assessments, the estimated fatty acid intakes may be prone to respondent bias and measurement error related to data included in food composition tables. However, as outlined above, we adopted several quality control measures for the matching procedure with the USDA database [42]. An additional limitation was that despite our comprehensive analyses according to tumour molecular subtypes we lacked data to examine the dietary TFA intake and breast cancer association for luminal A and luminal B tumours. Another possible limitation of our study is that dietary conjugated linoleic acid supplement use data was not collected so our analyses were limited to dietary intakes only.

## Conclusion

Our findings support the hypothesis that dietary intake of ITFAs, in particular elaidic acid, may increase breast cancer risk. Although we observed positive relationships for intake of both conjugated linoleic acid and palmitelaidic acid with breast cancer risk, the high correlation between these fatty acids means we were unable to differentiate these associations. Further mechanistic studies are needed to identify biological pathways that may underlie these associations. If our results are confirmed in future studies, the current EU and WHO limits for acceptable thresholds for industrial *trans* fats in foods may need to be revised to safeguard public health [31, 32]. However, given the results of our study, as well as the accumulating evidence of their deleterious effects on health, recommendations to limit as much as possible human consumption of industrial *trans* fats should be considered globally.

## Supplementary Information

**Additional file 1: Table S1.** Population characteristics. **Table S2.** Spearman rank correlations between dietary intakes of *trans* fatty acids. **Table S3.** Food group sources of predominant ruminant and industrial *trans* fatty acids. **Table S4.** Associations between dietary intake of *trans* fatty acids and breast cancer risk according to oestrogen receptor status. **Table S5.** Associations between dietary intake of *trans* fatty acids and breast cancer risk according to progesterone receptor status. **Table S6.** Associations between dietary intake of *trans* fatty acids and breast cancer risk according to human epidermal growth factor receptor 2 status. **Table S7.** Associations between dietary intake of *trans* fatty acids and breast cancer risk according to body mass index group. **Table S8.** Associations between dietary intake of *trans* fatty acids and breast cancer risk according to menopausal status. **Table S9.** Associations between dietary intake of total *trans* industrial and ruminant fatty acids and breast cancer risk after adjustment for total energy using the residuals method. **Table S10.** Associations between dietary intake of total *trans* industrial and ruminant fatty acids and breast cancer risk after mutual adjustment. **Table S11.** Associations between dietary intake of total *trans* industrial and ruminant fatty acids and breast cancer risk after adjustment for dietary intakes of saturated fatty acid, monounsaturated fatty acid, and polyunsaturated fatty acid. **Table S12.** Associations between dietary intake of total *trans* fatty acids and breast cancer risk adjusted for the World Cancer Research Fund and Mediterranean diet scores. **Table S13.** P for trend values for the associations between dietary intakes of *trans* fatty acids and breast cancer risk using the continuous variable and quintile-median approaches. **Figure 1.** Associations between dietary intake of total *trans* industrial fatty acids and breast cancer risk by country. **Figure 2.** Associations between dietary intake of total trans ruminant fatty acids and breast cancer risk by country.

## Data Availability

For information on how to submit an application for gaining access to EPIC data and/or biospecimens, please follow the instructions at http://epic.iarc.fr/access/index.php.

## Abbreviations

BMI: Body mass index
CIs: Confidence intervals
ENDB: EPIC Nutrient Database
EPIC: European Prospective Investigation into Cancer and Nutrition
ER−: Oestrogen receptor
EU: European Union
HER2: Human epidermal growth factor receptor 2
HRs: Hazard ratios
ITFAs: Industrial *trans* fatty acids
MUFA: Monounsaturated fatty acids
NNDSR: National Nutrient Database for Standard Reference of the United States
OR: Odds ratio
PUFA: Polyunsaturated fatty acids
RTFAs: Ruminant *trans* fatty acids
SFA: Saturated Fatty Acids
TFAs: *Trans* fatty acids
USDA: United States Department of Agriculture
VITAL: VITamins And Lifestyle
WCRF: World Cancer Research Fund
WHO: World Health Organization
