# Supplementary material for: Dietary intake of trans fatty acids and breast cancer risk in 9 European countries
Source: BMC Med. 2021 Mar 30;19:81. doi: 10.1186/s12916-021-01952-3 (PMC8008592; doi:10.1186/s12916-021-01952-3)
Supplement: Supplementary file 1 — Additional file 1: Table S1. Population characteristics. Table S2. Spearman rank correlations between dietary intakes of trans fatty acids. Table S3. Food group sources of predominant ruminant and industrial trans fatty acids. Table S4. Associations between dietary intake of trans fatty acids and breast cancer risk according to oestrogen receptor status. Table S5. Associations between dietary intake of trans fatty acids and breast cancer risk according to progesterone receptor status. Table S6. Associations between dietary intake of trans fatty acids and breast cancer risk according to human epidermal growth factor receptor 2 status. Table S7. Associations between dietary intake of trans fatty acids and breast cancer risk according to body mass index group. Table S8. Associations between dietary intake of trans fatty acids and breast cancer risk according to menopausal status. Table S9. Associations between dietary intake of total trans industrial and ruminant fatty acids and breast cancer risk after adjustment for total energy using the residuals method. Table S10. Associations between dietary intake of total trans industrial and ruminant fatty acids and breast cancer risk after mutual adjustment. Table S11. Associations between dietary intake of total trans industrial and ruminant fatty acids and breast cancer risk after adjustment for dietary intakes of saturated fatty acid, monounsaturated fatty acid, and polyunsaturated fatty acid. Table S12. Associations between dietary intake of total trans fatty acids and breast cancer risk adjusted for the World Cancer Research Fund and Mediterranean diet scores. Table S13. P for trend values for the associations between dietary intakes of trans fatty acids and breast cancer risk using the continuous variable and quintile-median approaches. Figure 1. Associations between dietary intake of total trans industrial fatty acids and breast cancer risk by country. Figure 2. Associations between dietary intake of total trans rumina [file 12916_2021_1952_MOESM1_ESM.docx]

# Additional File 1

**Table S1: Population characteristics**

|  | Non-cases  n= 305,366  Median (IQR) | Incident breast cancer cases  n=13,241  Median (IQR) |
| --- | --- | --- |
| Age at recruitment, years | 50.8 ( 44.8 – 57.2 ) | 52.7 ( 47.8 – 58.3 ) |
| Follow-up, years | 14.9 ( 13.9 – 16.5 ) | 8.1 ( 4.3 – 11.7 ) |
| Weight, kg | 63.8 ( 57.5 – 71.4 ) | 64.5 ( 58.0 – 72.0 ) |
| Height, cm | 163 ( 159 – 167 ) | 163 ( 159 – 167 ) |
| Body mass index (BMI), kg/m^2^ | 24.0 ( 21.8 – 26.9 ) | 24.1 ( 21.9 – 26.9 ) |
| Number of full-term pregnancies | 1.0 ( 1.0 – 1.0) | 1.0 ( 1.0 – 1.0 ) |
| Ever use oral contraceptives (%) |  |  |
| Yes | 58.6 | 59.1 |
| Age at first birth combinations (%) |  |  |
| Nulliparous | 15.5 | 14.2 |
| Age at first birth <30 (1-2 children) | 46.6 | 48.4 |
| Age at first birth <30 (≥3 children) | 26.1 | 23.9 |
| Age at first birth ≥30 | 11.7 | 13.6 |
| Ever use hormone replacement therapy for menopause (%) |  |  |
| Yes | 58.4 | 67.8 |
| Ever breastfed (%) |  |  |
| Yes | 72.1 | 72.2 |
| Menopausal status (%) |  |  |
| Premenopausal | 35.2 | 25 |
| Postmenopausal | 42.9 | 49.8 |
| Age at menopause, years | 48.6 ±4.9 | 49.2 ±4.8 |
| Alcohol intake (%) |  |  |
| None | 16.5 | 13.8 |
| >60 g/day | 7.8 | 10.5 |
| Total dietary energy intake, kcal/day | 1,938 ( 1,608 – 2,321) | 1,964 ( 1,637 – 2,343) |
| Education status (%) |  |  |
| None and primary school | 29.7 | 26.6 |
| Higher education | 23.6 | 24.7 |
| Physical activity (%) |  |  |
| Inactive | 14.8 | 16.8 |
| Active | 8.3 | 7.1 |
| Smoking status (%) |  |  |
| Never | 57.2 | 55.2 |
| Current | 19.8 | 19.8 |

*IQR= interquartile range.*

**Table S2: Spearman rank correlations between dietary intakes of *trans* fatty acids**

|  | Total  industrial *trans* fatty acids | Elaidic acid (18:1n-9/12) | Total  ruminant *trans* fatty acids | Palmitelaidic acid (16:1n-7t) | Conjugated linoleic acid | Vaccenic acid (18:1n7t) |
| --- | --- | --- | --- | --- | --- | --- |
| Total industrial *trans* fatty acids | 1 |  |  |  |  |  |
| Elaidic acid (18:1n-9/12) | - | 1 |  |  |  |  |
| Total ruminant *trans* fatty acids | 0.12 | 0.09 | 1 |  |  |  |
| Palmitelaidic acid (16:1n-7t) | 0.13 | 0.10 | - | 1 |  |  |
| Conjugated linoleic acid | 0.14 | 0.12 | - | 0.77 | 1 |  |
| Vaccenic acid (18:1n7t) | -0.18 | -0.19 | - | -0.02 | 0.1 | 1 |

**Table S3: Food group sources of conjugated linoleic fatty acid (a ruminant *trans* fatty acid) and elaidic acid (the predominant industrial *trans* fatty acid)**

| Food group | Conjugated linoleic acid (%) |  | Food groups | Elaidic acid (%) |
| --- | --- | --- | --- | --- |
| Dairy products | 36.40 |  | Fat | 51.10 |
| Fat | 28.90 |  | Dairy products | 13.22 |
| Meat and meat products | 9.80 |  | Meat and meat products | 8.86 |
| Cakes and biscuits | 8.20 |  | Cereal and cereal products | 5.36 |
| Cereal and cereal products | 5.30 |  | Condiments and sauces | 2.69 |
| Condiments and sauces | 3.60 |  | Potatoes and potatoes products | 1.79 |
| Potatoes and potatoes products | 2.20 |  |  |  |
| Egg and egg products | 2.00 |  |  |  |
| Sugar and confectionaries | 1.40 |  |  |  |
| Soups, bouillon | 1.20 |  |  |  |

**Table S4: Associations between dietary intake of *trans* fatty acids and breast cancer risk according to individual estrogen receptor status**

|  | | ER- | ER+ |  |
| --- | --- | --- | --- | --- |
|  |  | **HR (95% CI)** | **HR (95% CI)** | **P_heterogeneity_** |
|  | | **n= 1,666** | **n= 7,508** |  |
| Total industrial *trans* fatty acids^a^ | Q1 | 1 (reference) | 1 (reference) |  |
|  | Q2 | 1.05 (0.91 - 1.22) | 1.15 ( 1.07 - 1.23 ) |  |
|  | Q3 | 1.08 (0.92 - 1.26) | 1.15 ( 1.07 - 1.25 ) |  |
|  | Q4 | 1.15 ( 0.95 - 1.38 ) | 1.12 ( 1.03 - 1.23 ) |  |
|  | P trend | 0.15 | 0.015 | 0.80 |
|  |  |  |  |  |
| Elaidic acid | Q1 | 1 (reference) | 1 (reference) |  |
|  | Q2 | 1.04 ( 0.90 - 1.21 ) | 1.14 ( 1.07 - 1.23 ) |  |
|  | Q3 | 1.08 ( 0.92 - 1.28 ) | 1.16 ( 1.08 - 1.26 ) |  |
|  | Q4 | 1.16 ( 0.96 - 1.39 ) | 1.11 ( 1.02 - 1.22 ) |  |
|  | P trend | 0.12 | 0.018 | 0.68 |
|  |  |  |  |  |
| Total ruminant *trans* fatty acids^b^ | Q1 | 1 (reference) | 1 (reference) |  |
|  | Q2 | 1.05 ( 0.89 - 1.25 ) | 1.01 ( 0.93 - 1.09 ) |  |
|  | Q3 | 1.14 ( 0.95 - 1.37 ) | 1.05 ( 0.96 - 1.14 ) |  |
|  | Q4 | 1.10 ( 0.90 - 1.34 ) | 1.11 ( 1.02 - 1.22 ) |  |
|  | P trend | 0.32 | 0.008 | 0.81 |
|  |  |  |  |  |
| Palmitelaidic acid | Q1 | 1 (reference) | 1 (reference) |  |
|  | Q2 | 0.92 ( 0.78 - 1.07 ) | 1.03 ( 0.95 - 1.11 ) |  |
|  | Q3 | 0.98 ( 0.82 - 1.17 ) | 1.06 ( 0.97 - 1.15 ) |  |
|  | Q4 | 1.02 ( 0.85 - 1.22 ) | 1.09 ( 1.00 - 1.18 ) |  |
|  | P trend | 0.59 | 0.054 | 0.29 |
|  |  |  |  |  |
| Conjugated linoleic acid | Q1 | 1 (reference) | 1 (reference) |  |
|  | Q2 | 1.04 ( 0.88 - 1.24 ) | 1.00 ( 0.92 - 1.08 ) |  |
|  | Q3 | 1.14 ( 0.95 - 1.37 ) | 1.05 ( 0.96 - 1.14 ) |  |
|  | Q4 | 1.15 ( 0.94 - 1.40 ) | 1.12 ( 1.02 - 1.23 ) |  |
|  | P trend | 0.14 | 0.006 | 0.87 |
|  |  |  |  |  |
| Vaccenic acid | Q1 | 1 (reference) | 1 (reference) |  |
|  | Q2 | 1.00 ( 0.80 - 1.26 ) | 1.01 ( 0.90 - 1.13 ) |  |
|  | Q3 | 0.96 ( 0.78 - 1.19 ) | 1.08 ( 0.97 - 1.20 ) |  |
|  | Q4 | 0.93 ( 0.74 - 1.16 ) | 1.06 ( 0.95 - 1.18 ) |  |
|  | P trend | 0.41 | 0.16 | 0.02 |

*ER-=estrogen receptor negative; ER+=estrogen receptor positive; HR = hazard ratio; CI = confidence interval.*

*Stratified by study centre and age (in one-year categories), and adjusted for total energy intake (kcal/day; continuous), body mass index (kg/m2; continuous), height (cm; continuous), alcohol consumption (g/day; continuous), education level (none and primary, technical or professional and secondary, higher education), age at first birth and parity combined (nulliparous, first birth before age 30 years, 1-2 children; first birth before age 30 years,≥3 children; first birth ≥ 30 years), physical activity (inactive, moderately inactive, moderately active, and active), menopausal status(premenopausal, postmenopausal, perimenopausal, surgical postmenopausal bilateral ovariectomy), and smoking status (never, former, current smoker, unknown).  ^a^Total industrial trans fatty acids included 18:1n-9t, 18:2n-6tt; ^b^Total ruminant trans fatty acids included 16:1n-9t,18:1n-7t, conjugated linoleic acid.*

**Table S5: Associations between dietary intake of *trans* fatty acids and breast cancer risk according to individual progesterone receptors status**

|  | | PR- | PR+ |  |
| --- | --- | --- | --- | --- |
|  |  | **n= 2,602** | **n= 5,080** | **P_heterogeneity_** |
|  | | **HR (95%CI)** | **HR (95%CI)** |  |
| Total industrial *trans* fatty acids^a^ | Q1 | 1 (reference) | 1 (reference) |  |
|  | Q2 | 1.18 ( 1.06 - 1.33 ) | 1.12 ( 1.03 - 1.21 ) |  |
|  | Q3 | 1.16 ( 1.02 - 1.32 ) | 1.17 ( 1.06 - 1.28 ) |  |
|  | Q4 | 1.25 ( 1.07 - 1.45 ) | 1.14 ( 1.03 - 1.28 ) |  |
|  | P trend | 0.010 | 0.007 | 0.62 |
|  |  |  |  |  |
| Elaidic acid | Q1 | 1 (reference) | 1 (reference) |  |
|  | Q2 | 1.18 ( 1.05 - 1.32 ) | 1.11 ( 1.03 - 1.21 ) |  |
|  | Q3 | 1.15 ( 1.01 - 1.31 ) | 1.19 ( 1.08 - 1.30 ) |  |
|  | Q4 | 1.23 ( 1.06 - 1.43 ) | 1.14 ( 1.02 - 1.27 ) |  |
|  | P trend | 0.017 | 0.006 | 0.75 |
|  |  |  |  |  |
| Total ruminant *trans* fatty acids^b^ | Q1 | 1 (reference) | 1 (reference) |  |
|  | Q2 | 1.04 ( 0.91 - 1.20 ) | 1.02 ( 0.93 - 1.13 ) |  |
|  | Q3 | 1.15 ( 1.00 - 1.33 ) | 1.01 ( 0.90 - 1.12 ) |  |
|  | Q4 | 1.09 ( 0.93 - 1.28 ) | 1.11 ( 0.99 - 1.24 ) |  |
|  | P trend | 0.22 | 0.067 | 0.94 |
|  |  |  |  |  |
| Palmitelaidic acid | Q1 | 1 (reference) | 1 (reference) |  |
|  | Q2 | 0.95 ( 0.84 - 1.08 ) | 1.01 ( 0.92 - 1.11 ) |  |
|  | Q3 | 1.01 ( 0.88 - 1.16 ) | 1.07 ( 0.97 - 1.18 ) |  |
|  | Q4 | 1.01 ( 0.88 - 1.16 ) | 1.09 ( 0.98 - 1.21 ) |  |
|  | P trend | 0.63 | 0.058 | 0.35 |
|  |  |  |  |  |
| Conjugated linoleic acid | Q1 | 1 (reference) | 1 (reference) |  |
|  | Q2 | 1.08 ( 0.94 - 1.24 ) | 0.99 ( 0.90 - 1.10 ) |  |
|  | Q3 | 1.14 ( 0.98 - 1.32 ) | 1.01 ( 0.91 - 1.12 ) |  |
|  | Q4 | 1.17 ( 1.00 - 1.37 ) | 1.10 ( 0.98 - 1.24 ) |  |
|  | P trend | 0.055 | 0.057 | 0.66 |
|  |  |  |  |  |
| Vaccenic acid | Q1 | 1 (reference) | 1 (reference) |  |
|  | Q2 | 0.90 ( 0.76 - 1.07 ) | 1.07 ( 0.94 - 1.22 ) |  |
|  | Q3 | 0.93 ( 0.80 - 1.09 ) | 1.14 ( 1.01 - 1.28 ) |  |
|  | Q4 | 0.96 ( 0.82 - 1.14 ) | 1.07 ( 0.95 - 1.21 ) |  |
|  | P trend | 0.97 | 0.34 | 0.06 |

*PR-=progesterone receptor negative; PR+=progesterone receptor positive; HR = hazard ratio; CI = confidence interval.*

*Stratified by study centre and age (in one-year categories), and adjusted for total energy intake (kcal/day; continuous), body mass index (kg/m2; continuous), height (cm; continuous), alcohol consumption (g/day; continuous), education level (none and primary, technical or professional and secondary, higher education), age at first birth and parity combined (nulliparous, first birth before age 30 years, 1-2 children; first birth before age 30 years,≥3 children; first birth ≥ 30 years), physical activity (inactive, moderately inactive, moderately active, and active), menopausal status(premenopausal, postmenopausal, perimenopausal, surgical postmenopausal bilateral ovariectomy), and smoking status (never, former, current smoker, unknown).  ^a^Total industrial trans fatty acids included 18:1n-9t, 18:2n-6tt; ^b^Total ruminant trans fatty acids included 16:1n-9t,18:1n-7t, conjugated linoleic acid.*

**Table S6: Associations between dietary intake of *trans* fatty acids and molecular subtypes of breast cancer risk according to human epidermal growth factor receptor 2 (HER2) status**

|  | | ER- and PR- and HER2- | ER+ and PR+ and HER2+ | ER- and PR- and HER2+ | ER+ and PR+ and HER2- |  |
| --- | --- | --- | --- | --- | --- | --- |
|  |  | **n=412** | **n= 349** | **n=248** | **n=2,174** | **P_heterogeneity_** |
|  | | **HR (95%CI)** | **HR (95%CI)** | **HR (95%CI)** | **HR (95%CI)** |  |
| Total industrial *trans* fatty acids^a^ | Q1 | 1 (reference) | 1 (reference) | 1 (reference) | 1 (reference) |  |
|  | Q2 | 0.98 ( 0.73 - 1.31 ) | 0.77 ( 0.56 - 1.05 ) | 0.95 ( 0.65 - 1.39 ) | 1.18 ( 1.04 - 1.34 ) |  |
|  | Q3 | 1.00 ( 0.72 - 1.39 ) | 0.79 ( 0.55 - 1.14 ) | 0.98 ( 0.64 - 1.49 ) | 1.24 ( 1.08 - 1.44 ) |  |
|  | Q4 | 1.08 ( 0.74 - 1.58 ) | 0.78 ( 0.51 - 1.20 ) | 1.07 ( 0.66 - 1.74 ) | 1.16 ( 0.97 - 1.37 ) |  |
|  | P trend | 0.67 | 0.24 | 0.78 | 0.055 | 0.37 |
|  |  |  |  |  |  |  |
| Elaidic acid | Q1 | 1 (reference) | 1 (reference) | 1 (reference) | 1 (reference) |  |
|  | Q2 | 1.05 ( 0.78 - 1.41 ) | 0.73 ( 0.54 - 1.01 ) | 0.88 ( 0.60 - 1.30 ) | 1.16 ( 1.02 - 1.32 ) |  |
|  | Q3 | 1.02 ( 0.73 - 1.43 ) | 0.75 ( 0.52 - 1.07 ) | 1.02 ( 0.67 - 1.55 ) | 1.26 ( 1.09 - 1.45 ) |  |
|  | Q4 | 1.14 ( 0.78 - 1.67 ) | 0.73 ( 0.47 - 1.12 ) | 1.04 ( 0.64 - 1.69 ) | 1.15 ( 0.97 - 1.37 ) |  |
|  | P trend | 0.56 | 0.12 | 0.74 | 0.043 | 0.21 |
|  |  |  |  |  |  |  |
| Total ruminant *trans* fatty acids^b^ | Q1 | 1 (reference) | 1 (reference) | 1 (reference) | 1 (reference) |  |
|  | Q2 | 1.28 ( 0.91 - 1.80 ) | 0.96 ( 0.66 - 1.40 ) | 1.10 ( 0.68 - 1.76 ) | 1.06 ( 0.91 - 1.24 ) |  |
|  | Q3 | 1.15 ( 0.80 - 1.65 ) | 1.00 ( 0.67 - 1.48 ) | 1.49 ( 0.93 - 2.40 ) | 1.09 ( 0.93 - 1.28 ) |  |
|  | Q4 | 1.08 ( 0.72 - 1.62 ) | 0.88 ( 0.56 - 1.37 ) | 1.51 ( 0.90 - 2.53 ) | 1.23 ( 1.03 - 1.46 ) |  |
|  | P trend | 0.84 | 0.60 | 0.07 | 0.018 | 0.25 |
|  |  |  |  |  |  |  |
| Palmitelaidic acid | Q1 | 1 (reference) | 1 (reference) | 1 (reference) | 1 (reference) |  |
|  | Q2 | 0.68 ( 0.49 - 0.95 ) | 0.79 ( 0.55 - 1.15 ) | 0.90 ( 0.58 - 1.41 ) | 1.07 ( 0.92 - 1.24 ) |  |
|  | Q3 | 0.75 ( 0.53 - 1.05 ) | 0.98 ( 0.67 - 1.44 ) | 1.13 ( 0.70 - 1.82 ) | 1.06 ( 0.91 - 1.24 ) |  |
|  | Q4 | 0.79 ( 0.55 - 1.14 ) | 1.05 ( 0.69 - 1.60 ) | 1.27 ( 0.77 - 2.09 ) | 1.12 ( 0.94 - 1.32 ) |  |
|  | P trend | 0.39 | 0.45 | 0.21 | 0.27 | 0.43 |
|  |  |  |  |  |  |  |
| Conjugated linoleic acid | Q1 | 1 (reference) | 1 (reference) | 1 (reference) | 1 (reference) |  |
|  | Q2 | 1.20 ( 0.85 - 1.70 ) | 1.01 ( 0.70 - 1.46 ) | 0.92 ( 0.57 - 1.48 ) | 1.04 ( 0.89 - 1.21 ) |  |
|  | Q3 | 1.17 ( 0.81 - 1.67 ) | 1.04 ( 0.71 - 1.54 ) | 1.27 ( 0.79 - 2.03 ) | 1.10 ( 0.94 - 1.30 ) |  |
|  | Q4 | 1.05 ( 0.70 - 1.58 ) | 0.84 ( 0.53 - 1.32 ) | 1.52 ( 0.92 - 2.52 ) | 1.29 ( 1.08 - 1.54 ) |  |
|  | P trend | 0.99 | 0.47 | 0.041 | 0.002 | 0.11 |
|  |  |  |  |  |  |  |
| Vaccenic acid | Q1 | 1 (reference) | 1 (reference) | 1 (reference) | 1 (reference) |  |
|  | Q2 | 0.84 ( 0.53 - 1.35 ) | 0.93 ( 0.57 - 1.54 ) | 1.63 ( 0.83 - 3.20 ) | 1.14 ( 0.92 - 1.41 ) |  |
|  | Q3 | 0.73 ( 0.47 - 1.15 ) | 0.78 ( 0.48 - 1.26 ) | 1.96 ( 1.03 - 3.73 ) | 1.14 ( 0.93 - 1.40 ) |  |
|  | Q4 | 0.82 ( 0.53 - 1.29 ) | 0.93 ( 0.59 - 1.48 ) | 1.39 ( 0.73 - 2.64 ) | 1.08 ( 0.89 - 1.33 ) |  |
|  | P trend | 0.51 | 0.83 | 0.62 | 0.78 | 0.73 |

*ER-/PR-/HER2-=estrogen receptor negative/progesterone receptor negative/human epidermal growth factor receptor 2 negative; ER+/PR+/HER2+=estrogen receptor positive/progesterone receptor positive /human epidermal growth factor receptor 2 positive; ER-/PR-/HER2+=estrogen receptor negative/progesterone receptor negative /human epidermal growth factor receptor 2 positive; ER+/PR+/HER2-=estrogen receptor positive/progesterone receptor positive /human epidermal growth factor receptor 2 negative; HR = hazard ratio; CI = confidence interval*

*Stratified by study centre and age (in one-year categories), and adjusted for total energy intake (kcal/day; continuous), body mass index (kg/m2; continuous), height (cm; continuous), alcohol consumption (g/day; continuous), education level (none and primary, technical or professional and secondary, higher education), age at first birth and parity combined (nulliparous, first birth before age 30 years, 1-2 children; first birth before age 30 years,≥3 children; first birth ≥ 30 years), physical activity (inactive, moderately inactive, moderately active, and active), menopausal status(premenopausal, postmenopausal, perimenopausal, surgical postmenopausal bilateral ovariectomy), and smoking status (never, former, current smoker, unknown).  ^a^Total industrial trans fatty acids included 18:1n-9t, 18:2n-6tt; ^b^Total ruminant trans fatty acids included 16:1n-9t,18:1n-7t, conjugated linoleic acid.*

**Table S7: Associations between dietary intake of *trans* fatty acids and breast cancer risk according to body mass index group**

|  | | Normal weight  <25 kg/m^2^ | Overweight  25-<30 kg/m^2^ | Obese  ≥30 kg/m^2^ |  |
| --- | --- | --- | --- | --- | --- |
|  |  | **n= 7,801**  **HR (95%CI)** | **n=3,935**  **HR (95%CI)** | **n=1,505**  **HR (95%CI)** | **P_heterogeneity_** |
| Total industrial *trans* fatty acids^a^ | Q1 | 1 (reference) | 1 (reference) | 1 (reference) |  |
|  | Q2 | 1.08 ( 1.00 - 1.17 ) | 1.11 ( 0.99 - 1.24 ) | 1.20 ( 1.00 - 1.45 ) |  |
|  | Q3 | 1.07 ( 0.98 - 1.16 ) | 1.18 ( 1.04 - 1.33 ) | 1.27 ( 1.04 - 1.55 ) |  |
|  | Q4 | 1.10 ( 1.01 - 1.21 ) | 1.17 ( 1.03 - 1.32 ) | 1.31 ( 1.07 - 1.61 ) |  |
|  | Q5 | 1.09 ( 0.99 - 1.21 ) | 1.16 ( 1.01 - 1.33 ) | 1.27 ( 1.02 - 1.59 ) |  |
|  | P trend | 0.10 | 0.06 | 0.05 | 0.75 |
|  |  |  |  |  |  |
| Total ruminant *trans* fatty acids^b^ | Q1 | 1 (reference) | 1 (reference) | 1 (reference) |  |
|  | Q2 | 1.01 ( 0.93 - 1.10 ) | 1.02 ( 0.92 - 1.14 ) | 1.11 ( 0.94 - 1.32 ) |  |
|  | Q3 | 1.03 ( 0.94 - 1.12 ) | 0.97 ( 0.86 - 1.09 ) | 1.18 ( 0.98 - 1.43 ) |  |
|  | Q4 | 1.10 ( 1.00 - 1.20 ) | 0.99 ( 0.88 - 1.13 ) | 1.11 ( 0.90 - 1.37 ) |  |
|  | Q5 | 1.09 ( 0.99 - 1.20 ) | 1.04 ( 0.91 - 1.20 ) | 1.11 ( 0.88 - 1.41 ) |  |
|  | P trend | 0.02 | 0.71 | 0.45 | 0.85 |

*HR = hazard ratio; CI = confidence interval*

*Stratified by study centre and age (in one-year categories), and adjusted for total energy intake(kcal/day; continuous), height (cm; continuous), alcohol consumption (g/day; continuous), education level (none and primary, technical or professional and secondary, higher education), age at first birth and parity combined (nulliparous, first birth before age 30 years, 1-2 children; first birth before age 30 years,≥3 children; first birth ≥30 years), physical activity (inactive, moderately inactive, moderately active, and active), menopausal status (premenopausal, postmenopausal, perimenopausal, surgical postmenopausal bilateral ovariectomy), and smoking status (never, former, current, unknown).*

*^a^Total industrial trans fatty acids included 18:1n-9t, 18:2n-6tt; ^b^Total ruminant trans fatty acids included 16:1n-9t, 18:1n-7t, conjugated linoleic acid.*

**Table S8: Associations between dietary intake of *trans* fatty acids and breast cancer risk according to menopausal status**

|  | | Premenopausal | Postmenopausal |  |
| --- | --- | --- | --- | --- |
|  |  | **n=3,297**  **HR (95%CI)** | **n=6,592**  **HR (95%CI)** | **P_heterogeneity_** |
| Total industrial *trans* fatty acids^a^ | Q1 | 1 (reference) | 1 (reference) |  |
|  | Q2 | 1.11 ( 0.99 - 1.25 ) | 1.09 ( 0.98 - 1.22 ) |  |
|  | Q3 | 1.12 ( 0.98 - 1.27 ) | 1.08 ( 0.96 - 1.22 ) |  |
|  | Q4 | 1.09 ( 0.95 - 1.25 ) | 1.04 ( 0.91 - 1.18 ) |  |
|  | Q5 | 1.15 ( 0.99 - 1.34 ) | 1.10 ( 0.95 - 1.27 ) |  |
|  | P trend | 0.19 | 0.50 | 0.18 |
|  |  |  |  |  |
| Total ruminant *trans* fatty acids^b^ | Q1 | 1 (reference) | 1 (reference) |  |
|  | Q2 | 1.03 ( 0.90 - 1.18 ) | 1.05 ( 0.93 - 1.19 ) |  |
|  | Q3 | 1.04 ( 0.90 - 1.20 ) | 1.07 ( 0.93 - 1.23 ) |  |
|  | Q4 | 1.09 ( 0.94 - 1.27 ) | 1.11 ( 0.97 - 1.29 ) |  |
|  | Q5 | 1.10 ( 0.93 - 1.29 ) | 1.12 ( 0.96 - 1.30 ) |  |
|  | P trend | 0.21 | 0.13 | 0.79 |

*HR = hazard ratio; CI = confidence interval*

*Stratified by study centre and age (in one-year categories), and adjusted for total energy intake (kcal/day; continuous), body mass index (kg/m^2^; continuous), height (cm; continuous), alcohol consumption (g/day; continuous), educational level (none and primary, technical or professional and secondary, higher education), age at first birth and parity combined (nulliparous, first birth before age 30 years, 1-2 children; first birth before age 30 years,≥3 children; first birth after age 30 years), physical activity (inactive, moderately inactive, moderately active, and active), and smoking status (never, former, smoker, unknown).*

*^a^Total trans industrial fatty acids included 18:1n-9t, 18:2n-6tt; ^b^Total trans ruminant fatty acids included 16:1n-9t, 18:1n-7t, conjugated linoleic acid.*

**Table S9:** **Associations between dietary intake of total *trans* industrial and *trans* ruminant fatty acids and breast cancer risk after adjustment for total energy using the residuals method**

|  |  | Multivariable | Multivariable + Adjustment for total energy using the residual method |
| --- | --- | --- | --- |
|  |  | **HR (95%CI)** | **HR (95%CI)** |
| Total industrial *trans* fatty acids^a^ | Q1 | 1 (reference) | 1 (reference) |
|  | Q2 | 1.11 ( 1.04 - 1.18 ) | 1.08 ( 1.02 - 1.14 ) |
|  | Q3 | 1.12 ( 1.05 - 1.19 ) | 1.07 ( 1.02 – 1.14 ) |
|  | Q4 | 1.16 ( 1.08 - 1.24 ) | 1.09 ( 1.03 - 1.15 ) |
|  | Q5 | 1.14 ( 1.06 - 1.23 ) | 1.10 ( 1.04 - 1.17 ) |
|  | P trend | 0.001 | 0.001 |
|  |  |  |  |
| Total ruminant *trans* fatty acids^b^ | Q1 | 1 (reference) | 1 (reference) |
|  | Q2 | 1.02 ( 0.96 - 1.08 ) | 1.01 (0.96 - 1.07) |
|  | Q3 | 1.03 ( 0.96 - 1.10 ) | 1.03 (0.97 - 1.08) |
|  | Q4 | 1.08 ( 1.01 - 1.16 ) | 1.08 (1.02 - 1.14) |
|  | Q5 | 1.09 ( 1.01 - 1.17 ) | 1.06 (1.01 - 1.12) |
|  | P trend | 0.015 | 0.002 |

*HR = hazard ratio; CI = confidence interval*

*Stratified by study centre and age (in one-year categories), adjusted for total energy intake (kcal/day; continuous), body mass index (kg/m^2^; continuous), height (cm; continuous), alcohol consumption (g/day; continuous), education level (none and primary, technical or professional and secondary, higher education), age at first birth and parity combined (nulliparous, first birth before age 30 years, 1-2 children; first birth before age 30 years,≥3 children; first birth ≥ 30 years), physical activity (inactive, moderately inactive, moderately active, and active), menopausal status (premenopausal, postmenopausal, perimenopausal, surgical postmenopausal bilateral ovariectomy), and smoking status (never, former, current, unknown), and for total energy intake using the residual method.*

*^a^Total industrial trans fatty acids included 18:1n-9t, 18:2n-6tt; ^b^Total ruminant trans fatty acids included16:1n-9t, 18:1n-7t, conjugated linoleic acid.*

**Table S10: Associations between dietary intake of total *trans* industrial and *trans* ruminant fatty acids and breast cancer risk after mutual adjustment**

|  | | Multivariable | Multivariable  + RTFA | Multivariable  +ITFA |
| --- | --- | --- | --- | --- |
|  |  | **HR (95%CI)** | **HR (95%CI)** | **HR (95%CI)** |
| Total industrial *trans* fatty acids^a^ | Q1 | 1 (reference) | 1 (reference) | - |
|  | Q2 | 1.11 ( 1.04 - 1.18 ) | 1.10 ( 1.03-1.17 ) | - |
|  | Q3 | 1.12 ( 1.05 - 1.19 ) | 1.11 ( 1.04-1.19 ) | - |
|  | Q4 | 1.16 ( 1.08 - 1.24 ) | 1.13 ( 1.06-1.22 ) | - |
|  | Q5 | 1.14 ( 1.06 - 1.23 ) | 1.13 ( 1.04-1.22 ) | - |
|  | P trend | 0.001 | 0.006 | - |
|  |  |  |  |  |
| Total ruminant *trans* fatty acids^b^ | Q1 | 1 (reference) | - | 1 (reference) |
|  | Q2 | 1.02 ( 0.96 - 1.08 ) | - | 1.03 ( 0.97-1.10 ) |
|  | Q3 | 1.03 ( 0.96 - 1.10 ) | - | 1.03 ( 0.96-1.10 ) |
|  | Q4 | 1.08 ( 1.01 - 1.16 ) | - | 1.07 ( 1.00-1.15 ) |
|  | Q5 | 1.09 ( 1.01 - 1.17 ) | - | 1.07 ( 0.99-1.15 ) |
|  | P trend | 0.015 | - | 0.060 |

*HR = hazard ratio; CI = confidence interval*

*Stratified by study centre and age (in one-year categories), adjusted for total energy intake (kcal/day; continuous), body mass index (kg/m^2^; continuous), height (cm; continuous), alcohol consumption (g/day; continuous), education level (none and primary, technical or professional and secondary, higher education), age at first birth and parity combined (nulliparous, first birth before age 30 years, 1-2 children; first birth before age 30 years,≥3 children; first birth ≥ 30 years), physical activity (inactive, moderately inactive, moderately active, and active), menopausal status (premenopausal, postmenopausal, perimenopausal, surgical postmenopausal bilateral ovariectomy), and smoking status (never, former, current, unknown), and mutual adjustments for total trans industrial (ITFA) and trans ruminant fatty acids(RTFA)(continuous).*

*^a^Total industrial trans fatty acids included 18:1n-9t, 18:2n-6tt; ^b^Total ruminant trans fatty acids included16:1n-9t, 18:1n-7t, conjugated linoleic acid.*

**Table S11: Associations between dietary intake of total *trans* industrial and *trans* ruminant fatty acids and breast cancer risk after adjustment for dietary intakes of saturated fatty acid (SFA), monounsaturated fatty acid (MUFA), and polyunsaturated fatty acid (PUFA)**

|  | | Multivariable | Multivariable +  SFA | Multivariable +  MUFA | Multivariable +  PUFA |
| --- | --- | --- | --- | --- | --- |
|  |  | **HR (95%CI)** | **HR (95%CI)** | **HR (95%CI)** | **HR (95%CI)** |
| Total industrial *trans* fatty acids^a^ | Q1 | 1 (reference) | 1 (reference) | 1 (reference) | 1 (reference) |
|  | Q2 | 1.11 ( 1.04 - 1.18 ) | 1.10 ( 1.04-1.17 ) | 1.10 ( 1.04-1.17 ) | 1.11 ( 1.04-1.17 ) |
|  | Q3 | 1.12 ( 1.05 - 1.19 ) | 1.12 ( 1.05-1.19 ) | 1.12 ( 1.05-1.19 ) | 1.12 ( 1.05-1.20 ) |
|  | Q4 | 1.16 ( 1.08 - 1.24 ) | 1.14 ( 1.07-1.23 ) | 1.14 ( 1.07-1.23 ) | 1.15 ( 1.07-1.23 ) |
|  | Q5 | 1.14 ( 1.06 - 1.23 ) | 1.13 ( 1.05-1.22 ) | 1.13 ( 1.05-1.22 ) | 1.15 ( 1.06-1.24 ) |
|  | P trend | 0.001 | 0.004 | 0.004 | 0.001 |
|  |  |  |  |  |  |
| Total ruminant *trans* fatty acids^b^ | Q1 | 1 (reference) | 1 (reference) | 1 (reference) | 1 (reference) |
|  | Q2 | 1.02 ( 0.96 - 1.08 ) | 1.03 ( 0.97-1.09 ) | 1.03 ( 0.97-1.10 ) | 1.03 ( 0.97-1.10 ) |
|  | Q3 | 1.03 ( 0.96 - 1.10 ) | 1.03 ( 0.96-1.10 ) | 1.03 ( 0.96-1.10 ) | 1.03 ( 0.97-1.10 ) |
|  | Q4 | 1.08 ( 1.01 - 1.16 ) | 1.07 ( 0.99-1.15 ) | 1.08 ( 1.00-1.15 ) | 1.08 ( 1.01-1.15 ) |
|  | Q5 | 1.09 ( 1.01 - 1.17 ) | 1.08 ( 0.99-1.17 ) | 1.08 ( 1.00-1.17 ) | 1.09 ( 1.01-1.17 ) |
|  | P trend | 0.015 | 0.042 | 0.026 | 0.015 |

*HR = hazard ratio; CI = confidence interval*

*Stratified by study centre and age (in one-year categories), adjusted for total energy intake (kcal/day; continuous), body mass index (kg/m^2^; continuous), height (cm; continuous), alcohol consumption (g/day; continuous), education level (none and primary, technical or professional and secondary, higher education), age at first birth and parity combined (nulliparous, first birth before age 30 years, 1-2 children; first birth before age 30 years,≥3 children; first birth ≥ 30 years), physical activity (inactive, moderately inactive, moderately active, and active), menopausal status (premenopausal, postmenopausal, perimenopausal, surgical postmenopausal bilateral ovariectomy), and smoking status (never, former, current, unknown), and mutual adjustments for total trans industrial and trans ruminant fatty acids as well as adjustment for saturated fatty acid (SFA), monounsaturated fatty acid (MUFA) and polyunsaturated fatty acid (PUFA) (continuous).*

*^a^Total industrial trans fatty acids included 18:1n-9t, 18:2n-6tt; ^b^Total ruminant trans fatty acids included16:1n-9t, 18:1n-7t, conjugated linoleic acid.*

**Table S12: Associations between dietary intake of total *trans* fatty acids and breast cancer risk adjusted for the World Cancer Research Fund (WCRF) and Mediterranean diet scores**

|  | | Multivariable  HR (95%CI) | Multivariable + WCRF diet score HR (95%CI) | Multivariable + Mediterranean diet score  HR (95%CI) |
| --- | --- | --- | --- | --- |
| Total industrial *trans* fatty acids^a^ | Q1 | 1 (reference) | 1 (reference) | 1 (reference) |
|  | Q2 | 1.11 ( 1.04 - 1.18 ) | 1.10 ( 1.03 - 1.17 ) | 1.10 ( 1.04 - 1.17 ) |
|  | Q3 | 1.12 ( 1.05 - 1.19 ) | 1.11 ( 1.03 - 1.19 ) | 1.12 ( 1.05 - 1.19 ) |
|  | Q4 | 1.16 ( 1.08 - 1.24 ) | 1.14 ( 1.06 - 1.23 ) | 1.14 ( 1.07 - 1.22 ) |
|  | Q5 | 1.14 ( 1.06 - 1.23 ) | 1.14 ( 1.05 - 1.24 ) | 1.13 ( 1.05 - 1.22 ) |
|  | P trend | 0.001 | 0.002 | 0.004 |
|  |  |  |  |  |
| Total ruminant *trans* fatty acids^b^ | Q1 | 1 (reference) | 1 (reference) | 1 (reference) |
|  | Q2 | 1.02 ( 0.96 - 1.08 ) | 1.05 ( 0.97 - 1.13 ) | 1.03 ( 0.97 - 1.10 ) |
|  | Q3 | 1.03 ( 0.96 - 1.10 ) | 1.05 ( 0.97 - 1.13 ) | 1.03 ( 0.96 - 1.10 ) |
|  | Q4 | 1.08 ( 1.01 - 1.16 ) | 1.08 ( 0.99 - 1.16 ) | 1.08 ( 1.01 - 1.16 ) |
|  | Q5 | 1.09 ( 1.01 - 1.17 ) | 1.09 ( 1.00 - 1.18 ) | 1.09 ( 1.01 - 1.17 ) |
|  | P trend | 0.015 | 0.063 | 0.019 |

*Stratified by study centre and age (in one-year categories), and adjusted for total energy intake (kcal/day; continuous), body mass index (kg/m^2^; continuous), height (cm; continuous), alcohol consumption (g/day; continuous), educational level (none and primary, technical or professional and secondary, higher education), age at first birth and parity combined (nulliparous, first birth before age 30 years, 1-2 children; first birth before age 30 years,≥3 children; first birth after age 30 years), physical activity (inactive, moderately inactive, moderately active, and active), and smoking status (never, former, smoker, unknown) and for the WCRF or Mediterranean diet scores ( quintiles).*

*^a^Total trans industrial fatty acids included 18:1n-9t, 18:2n-6tt; ^b^Total trans ruminant fatty acids included 16:1n-9t, 18:1n-7t, conjugated linoleic acid.*

**Table S13: P for trend values for the associations between dietary intakes of *trans* fatty acids and breast cancer risk using the continuous variable and quintile-median approaches**

|  | Continuous variable P trend | Quintile-median  P trend |
| --- | --- | --- |
| Total industrial *trans* fatty acids^a^ | 0.001 | 0.05 |
| Elaidic acid | 0.001 | 0.045 |
| Total ruminant *trans* fatty acids^b^ | 0.015 | 0.031 |
| Palmitelaidic acid | 0.028 | 0.05 |
| Conjugated linoleic acid | 0.001 | 0.007 |
| Vaccenic Acid | 0.51 | 0.91 |

*Stratified by study centre and age (in one-year categories), and adjusted for total energy intake (kcal/day; continuous), body mass index (kg/m^2^; continuous), height (cm; continuous), alcohol consumption (g/day; continuous), educational level (none and primary, technical or professional and secondary, higher education), age at first birth and parity combined (nulliparous, first birth before age 30 years, 1-2 children; first birth before age 30 years,≥3 children; first birth after age 30 years), physical activity (inactive, moderately inactive, moderately active, and active), and smoking status (never, former, smoker, unknown).*

*^a^Total trans industrial fatty acids included 18:1n-9t, 18:2n-6tt; ^b^Total trans ruminant fatty acids included 16:1n-9t, 18:1n-7t, conjugated linoleic acid.*

*Stratified by study centre and age (in one-year categories), and adjusted for total energy intake(kcal/day; continuous), body mass index (kg/m^2^; continuous), height (cm; continuous), alcohol consumption (g/day; continuous), education level (none and primary, technical or professional and secondary, higher education), age at first birth and parity combined (nulliparous, first birth before age 30 years, 1-2 children; first birth before age 30 years,≥3 children; first birth ≥30 years ), physical activity (inactive, moderately inactive, moderately active, and active), menopausal status(premenopausal, postmenopausal, perimenopausal, surgical postmenopausal bilateral ovariectomy), and smoking status (never, former, current smoker, unknown).*

Figure S1: Associations between dietary intake of total *trans* industrial fatty acids and breast cancer risk by country

*Stratified by study centre and age (in one-year categories), and adjusted for total energy intake(kcal/day; continuous), body mass index (kg/m^2^; continuous), height (cm; continuous), alcohol consumption (g/day; continuous), education level (none and primary, technical or professional and secondary, higher education), age at first birth and parity combined (nulliparous, first birth before age 30 years, 1-2 children; first birth before age 30 years,≥3 children; first birth ≥30 years ), physical activity (inactive, moderately inactive, moderately active, and active), menopausal status(premenopausal, postmenopausal, perimenopausal, surgical postmenopausal bilateral ovariectomy), and smoking status (never, former, current smoker, unknown).*

Figure S2: Associations between dietary intake of total *trans* ruminant fatty acids and breast cancer risk by country
